# Partial Nuclear Extrusion in Chronic Lymphocytic Leukemia Observed to Be an In Vitro Artefact

**DOI:** 10.1002/ajh.70041

**Published:** 2025-08-22

**Authors:** Rima Chatila, Guillaume Beziat, Laure Pirovano, Clément Bouyssie, Jean‐Baptiste Rieu, Barbara J. Bain

**Affiliations:** ^1^ Medical Biology Laboratory Albi General Hospital Albi France; ^2^ Department of Haematology Albi General Hospital Albi France; ^3^ Haematology Laboratory, University Hospital of Toulouse Cancer University Institute of Toulouse–Oncopole Toulouse France; ^4^ Centre for Haematology, St Mary's Hospital Campus of Imperial College Faculty of Medicine, St Mary's Hospital London UK

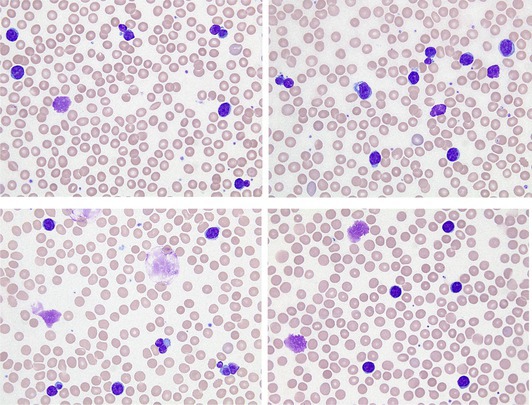

A 69‐year‐old man with a 10‐year history of untreated chronic lymphocytic leukemia (CLL) was referred for his annual hematology follow‐up. He was in good general condition (ECOG 1), with no lymphadenopathy or splenomegaly on physical examination. His blood count showed: hemoglobin 125 g/L, platelet count 217 × 10^9^/L, and white blood cell count 66.96 × 10^9^/L with a marked lymphocytosis (94%, 62.94 × 10^9^/L). Blood film examination revealed a monomorphic population of small lymphocytes and smudge cells with typical features of CLL. However, 15% of lymphocytes displayed partial nuclear extrusion (upper images, all images May‐Grünwald‐Giemsa, ×63 objective), a finding already observed in this patient's previous blood films.

To assess whether this morphological abnormality occurred in vivo, a blood film was directly performed at the patient's bedside using a non‐anticoagulated sample, in parallel with blood samples collected in EDTA and lithium heparin tubes. Strikingly, nuclear extrusion was clearly observed in the blood films from both anticoagulated samples, EDTA (top) and lithium heparin (bottom left), but was completely absent in the non‐anticoagulated blood film (bottom right). These findings indicate that, in this case, nuclear extrusion is an in vitro phenomenon occurring in anticoagulated blood.

A few cases of nuclear extrusion in CLL lymphocytes have been reported in the literature [1, 2, 3], but the underlying mechanism remains unclear. Some authors have argued against an in vitro artefact, citing the consistent presence of this morphological feature in repeated samples from the same patient [2]. However, those observations were limited to heparin‐anticoagulated samples. Other authors have suggested that nuclear extrusion reflects the intrinsic fragility of CLL lymphocytes [3]. Our findings challenge this interpretation: in our case, smudge cells (widely recognized as a marker of lymphocyte fragility, resulting from mechanical disruption during film preparation) were present in both anticoagulated and non‐anticoagulated blood samples. In contrast, nuclear extrusion was only observed in the anticoagulated samples. This discrepancy calls into question the hypothesis that nuclear extrusion is related to intrinsic cellular fragility.

Although the number of reported cases remains too limited to draw firm conclusions, our observation supports the hypothesis that this rare phenomenon only occurs in vitro, developing in anticoagulated blood, and does not appear to have any clinical impact on CLL progression. Moreover, it highlights the importance of performing a non‐anticoagulated blood film at the patient's bedside when such atypical morphological findings are observed.

## Conflicts of Interest

The authors declare no conflicts of interest.

## Data Availability

Data sharing is not applicable to this article.

## References

[1] D. G. Newell , U. Jayaswal , J. Smith , and S. Roath , “Unusual Lymphocyte Morphology in a Case of Chronic Lymphatic Leukaemia: Apparent Nuclear Extrusion,” Acta Haematologica 59, no. 1 (1978): 25–30. 414503 10.1159/000207741

[2] M. Djaldetti , Z. Malik , and U. Lewinski , “Ultrastructural and Functional Studied on the Lymphocytes of a Patient With Chronic Lymphocytic Leukaemia and Nuclear Extrusion,” Scandinavian Journal of Haematology 23, no. 5 (1979): 393–402. 94456 10.1111/j.1600-0609.1979.tb02739.x

[3] B. Z. Katz and Y. Herishanu , “Fragility of Sub‐Cellular Structures in Chronic Lymphocytic Leukemia,” International Journal of Hematology 105, no. 6 (2017): 707–708. 28324280 10.1007/s12185-017-2218-0

